# Charge-density-wave order takes over antiferromagnetism in Bi_2_Sr_2−*x*_La_*x*_CuO_6_ superconductors

**DOI:** 10.1038/s41467-017-01465-9

**Published:** 2017-11-02

**Authors:** S. Kawasaki, Z. Li, M. Kitahashi, C. T. Lin, P. L. Kuhns, A. P. Reyes, Guo-qing Zheng

**Affiliations:** 1 Department of Physics, Okayama University, Okayama, 700-8530 Japan; 2 Institute of Physics, Chinese Academy of Sciences, and Beijing National Laboratory for Condensed Matter Physics, 100190 Beijing, China; 3 Max-Planck-Institut fur Festkorperforschung, Heisenbergstrasse 1, D-70569 Stuttgart, Germany; 4 National High Magnetic Field Laboratory, Tallahassee, FL 32310 USA

## Abstract

Superconductivity appears in the cuprates when a spin order is destroyed, while the role of charge is less known. Recently, charge density wave (CDW) was found below the superconducting dome in YBa_2_Cu_3_O_*y*_ when a high magnetic field is applied perpendicular to the CuO_2_ plane, which was suggested to arise from incipient CDW in the vortex cores that becomes overlapped. Here by ^63^Cu-nuclear magnetic resonance, we report the discovery of CDW induced by an in-plane field, setting in above the dome in single-layered Bi_2_Sr_2−*x*_La_*x*_CuO_6_. The onset temperature *T*_CDW_ takes over the antiferromagnetic order temperature *T*_N_ beyond a critical doping level at which superconductivity starts to emerge, and scales with the pseudogap temperature T*. These results provide important insights into the relationship between spin order, CDW and the pseudogap, and their connections to high-temperature superconductivity.

## Abstract (web summary)

Whilst superconductivity usually appears when magnetic order is suppressed, the role of charge is less known. Here, Kawasaki et al. report a charge density wave (CDW) above the superconducting transition induced by an in-plane magnetic field in Bi_2_Sr_2-*x*_La_*x*_CuO_6_, with the CDW onset temperature scaling with the pseudogap temperature.

## Introduction

High transition-temperature (*T*_c_) superconductivity is obtained by doping carriers to destroy an antiferromagnetic (AF) spin ordered Mott insulating phase. Although it is generally believed that the interaction responsible for the spin order is important for the superconductivity^1^, the electron pairing mechanism is still elusive. This is because the nature of the normal state is still unclear^2,3^. For example, in the region with low carrier concentration *p* (0 < *p* < 0.2), a pseudogap state emerges where partial density of states (DOS) is lost below a characteristic temperature T* well above *T*_c_^4^ or even *T*_N_^5^. Although the nature of the strange metallic state is still under debate, it is likely connected to both spin and change fluctuations or even orders. In fact, experimental progress suggests that the spin and charge degrees of freedom are highly entangled.

For example, a striped spin/charge order was found around *x* ~ 1/8 in La_1.6−*x*_Nd_0.4_Sr_*x*_CuO_4_ (LSCO) two decades ago^6^. More recently, various forms of charge order were reported in many other systems. Scanning tunneling microscopy (STM) in Bi_2_Sr_2_CaCu_2_O_8+*δ*′_ found a modulation of local DOS in the vortex cores where superconductivity is destroyed^7^, which was interpreted as due to halos of incipient CDW localized within the cores^8,9^. Resonant elastic and inelastic x-ray spectroscopy (RXS) measurements found a short-range CDW with ordering vectors along the in-plane Cu-O bond directions, **q** = (~0.3, 0) and (0, ~0.3). The correlation length is $\xi _{a,b}$ ~ 50 Å for YBa_2_Cu_3_O_7−*y*_ (YBCO)^10,11^ and $\xi _{a,b}$ ~ 20 Å for the other systems^12–16^. Quite recently, it was suggested by ^17^O nuclear magnetic resonance (NMR) in YBCO that such CDW is of static origin^17^. In Bi_2_Sr_2−*x*_La_*x*_CuO_6+*δ*_ (Bi2201), most intriguingly, the onset temperature of the short-range CDW was found to coincide^12,16^ with T* that is far above *T*_c_ or *T*_N_^5,18^.

Application of a high magnetic field is useful to diagnose the interplay between various orders in the cuprates. When a high magnetic field is applied perpendicular to the CuO_2_ plane, superconductivity can be suppressed substantially. In YBCO, ^63^Cu NMR at *H* = 28.5 T revealed a long-range charge density modulation perpendicular to the CuO-chain in the sample with *p* = 0.108^19^. RXS also indicated that a high field induces a correlation along the CuO-chain direction and modifies the coupling between CuO_2_ bilayers, thus causes a three-dimensional CDW^20,21^. These observations are consistent with early discovery of a Fermi-surface reconstruction by quantum oscillations^22^ and a recent report of a thermodynamic phase transition^23^.

These findings have arisen much interests, but the origin of the CDW and its connection to superconductivity is yet unknown. As the long-range CDW onsets below *T*_c_(*H* = 0) and only emerges when the field is applied perpendicular to the CuO_2_ plane, a wide-spread speculation is that it is due to incipient CDW in the vortex cores^7^ that becomes overlapped as the field gets stronger^11,13,24^. In fact, a field as large as 28.5 T applied in the CuO_2_—plane of YBCO did not bring about any long-range CDW^19^. Also, the role of the CuO chain is unclear; in Bi_2_Sr_2_CaCu_2_O_8+*δ*_ without a CuO chain, no long-range CDW was found^25^.

In order to clarify the relationship between the intertwined AF spin order, CDW, pseudogap and superconductivity, we apply high magnetic fields up to 42.5 T parallel to the Cu−O bond direction (*H*||*a* or *b* axis) in Bi_2_Sr_2−*x*_La_*x*_CuO_6+*δ*_ where the pseudogap spans from the parent AF insulating phase to the overdoped superconducting regime^5,18^. This material has no CuO chain and the application of an in-plane field does not create vortex cores in the CuO_2_ plane. Surprisingly, we discover a long-range CDW that emerges far above the superconducting dome for *H*_||_ > 10 T. We find that such CDW order becomes the successor of the AF order beyond *p* = 0.107 at which superconductivity starts to emerge. The *T*_CDW_ takes over *T*_N_, but disappears well before the pseudogap closes. Our results indicate that CDW can be well disentangled from other orders.

## Results

### Evidence for a field-induced CDW in underdoped Bi2201

**Fig. 1:**
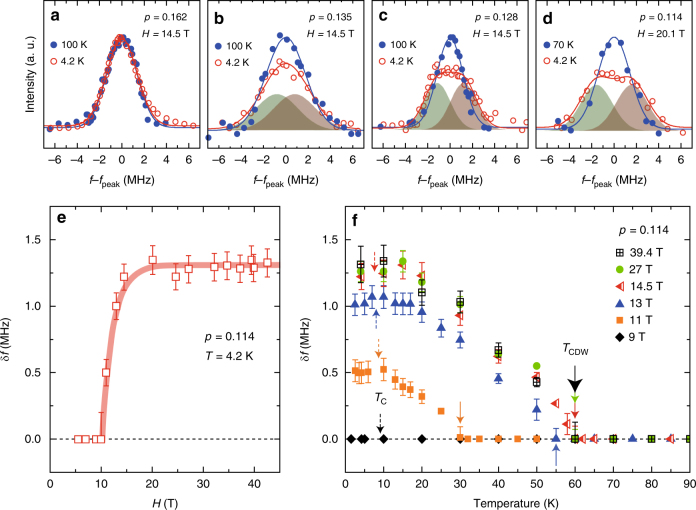
Field and temperature evolution of NMR satellite line for underdoped Bi2201. **a**–**d** The NMR satellite (3/2 ↔ 1/2 transition) lines for Bi_2_Sr_2−*x*_La_*x*_CuO_6+*δ*_ with *x* = 0.4 (*p* = 0.162), *x* = 0.6 (*p* = 0.135), *x* = 0.65 (*p* = 0.128), and *x* = 0.75 (*p* = 0.114). The curves for *p* = 0.135, 0.128, and 0.114 at *T* = 4.2 K are the sum of two Gaussian functions (shaded area). *f*_peak_ is the peak frequency. The intensity is normalized by the area of the spectrum. **e** Field evolution of the line splitting *δf* for *p* = 0.114 at *T* = 4.2 K. **f** Temperature evolution of *δf* for *p* = 0.114 under various fields. The solid arrows indicate *T*_CDW_. The dotted arrows indicate *T*_c_(*H*). Error bars represent the uncertainty in estimating *δf*

Figure 1a–d shows the ^63^Cu-NMR satellite (3/2 ↔ 1/2 transition) lines for four compounds of Bi_2_Sr_2−*x*_La_*x*_CuO_6+*δ*_ at two representative temperatures at *H*_||_ = 14.5 or 20.1 T. As seen in Fig. 1a, no change between *T* = 100 and 4.2 K is observed for the optimally doped compound (*p* = 0.162). However, the spectrum is broadened at *T* = 4.2 K for *p* = 0.135 (Fig. 1b), and a splitting ±*δf* of the spectrum is observed at *T* = 4.2 K for *p* = 0.128 and 0.114, as seen in Fig. 1c, d. The spectra at *T* = 4.2 K for *p* = 0.128 and 0.114 can be reproduced by a sum of two Gaussian functions. It is noted that at low fields below 10 T, the spectrum shows no appreciable temperature dependence in the whole temperature range. The NMR line splitting indicates a long-range order, as it measures an assemble of nuclear spins over the sample.

Figure 1e shows the field evolution of *δf* for *p* = 0.114. The *δf* grows steeply at *H*_||_ = 10.4 T and saturates above *H*_||_ ~ 14.5 T. Figure 1f shows the temperature dependence of *δf* for *p* = 0.114 under various fields. The *δf* grows rapidly below *T* ~ 30, 55, and 60 K at *H*_||_ = 11, 13, and above 14.5 T, respectively. These results indicate that a field-induced phase transition occurs in the underdoped Bi2201. The results are qualitatively similar to the results found in YBCO where the same transition line splits into two peaks due to the spatial modulation of the NQR frequency *ν*_Q_^19^.

**Fig. 2:**
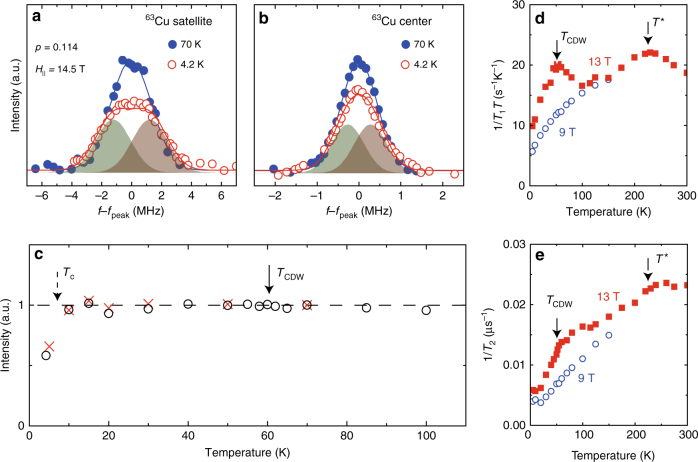
Evidence for the CDW without a spin order. Temperature dependence of the NMR satellite **a** and center **b** lines for *p* = 0.114 at *H* = 14.5 T. Solid curves are the results of Gaussian fittings. The curves at *T* = 4.2 K are the sum of two Gaussian functions (shaded area). **c** Temperature dependence of NMR signal intensity for the satellite line (open circles) and the center line (crosses). The intensity is corrected by taking into account the 1/*T* factor and the *T*_2_ effect. The absence of any intensity loss across *T*_CDW_ rules out the presence of any kinds of spin order. The intensity loss below *T*_c_ is due to the Meissner effect. The temperature dependencies of 1/*T*_1_ *T* **d** and 1/*T*_2_ **e** at different fields

Next we show that the field-induced phase transition is due to a charge order, but not spin order. Figure 2a, b, respectively, shows the temperature dependence of the satellite and the center lines for the sample with the lowest doping *p* = 0.114. A spectrum broadening is also found in the center line (1/2 ↔ −1/2 transition) at *T* = 4.2 K, but it is much smaller than the satellite line. Figure 2c shows the temperature dependence of the NMR intensity obtained by integrating the spectrum at each temperature. The intensity has no anomaly above *T*_c_, indicating that there is no spin order. For antiferromagnetic insulator *p* = 0.107 (*x* = 0.8), the intensity decreases below *T*_N_ = 66 K because an internal field shifts the peak frequency far away^5^. Furthermore, a possibility of striped phase formation leading to a wipe out effect found in LSCO^26^ can also be ruled out. It is noted that the possibility of a field-induced spin-density-wave order has already been ruled out previously for *p* = 0.162^27^.

Therefore, the splitting of the satellite line (Fig. 2a) and the broadening of the center line (Fig. 2b) is due to a distribution of the Knight shift $K_\parallel \pm \delta K_\parallel$ and the NQR frequency as *ν*_Q_ ± *δν* as observed in YBCO. Furthermore, the splitting *δf*_satellite_ = 1.22 MHz is much larger than *δf*_center_ = 0.271 MHz, indicating that the *ν*_Q_ change is the main contributor to the observed line splitting. By a simple calculation (Supplementary Note 1), we find that $\delta K_\parallel \sim 0.05 \pm 0.01\%$ and *δν* = 2.5 ± 0.2 MHz can reproduce both the satellite and the center lines at the same time (shaded areas in Fig. 2a, b). The relation *ν*_Q_ = 22.0 + 39.6 *p* (Supplementary Fig. 1) then yields a hole-concentration distribution $\delta p\sim 0.06 \pm 0.01$ at the Cu site. Since there is no spin order here (Fig. 2c) as mentioned already, the splitting of the satellite line *δf* (∝ *δp*) indicates a field-induced long-range charge distribution, i.e., a formation of CDW at low temperature in underdoped Bi2201.

Figure 2d, e shows the temperature dependence of the nuclear spin-lattice relaxation rate divided by *T*, 1/*T*_1_ *T* and spin-spin relaxation rate 1/*T*_2_ for *p* = 0.114 obtained at two different fields. At *H* = 9 T, both quantities decrease monotonically below T* = 230 K. At *H* = 13 T, however, a pronounced peak was found in 1/*T*_1_ *T* at *T*_CDW_ = 55 K. Such a peak in 1/*T*_1_ *T* is a characteristic of a CDW order^28^. The 1/*T*_2_ also shows a sharp decrease at *T*_CDW_ = 55 K. These results provide further evidence for a field-induced CDW phase transition.

### *H-T* phase diagram for underdoped Bi2201

To obtain the CDW onset temperature (*T*_CDW_) and the threshold field (*H*_CDW_) for *p* = 0.114, we study the temperature dependence of the NMR spectra at various magnetic fields (Fig. 1e, f).

**Fig. 3:**
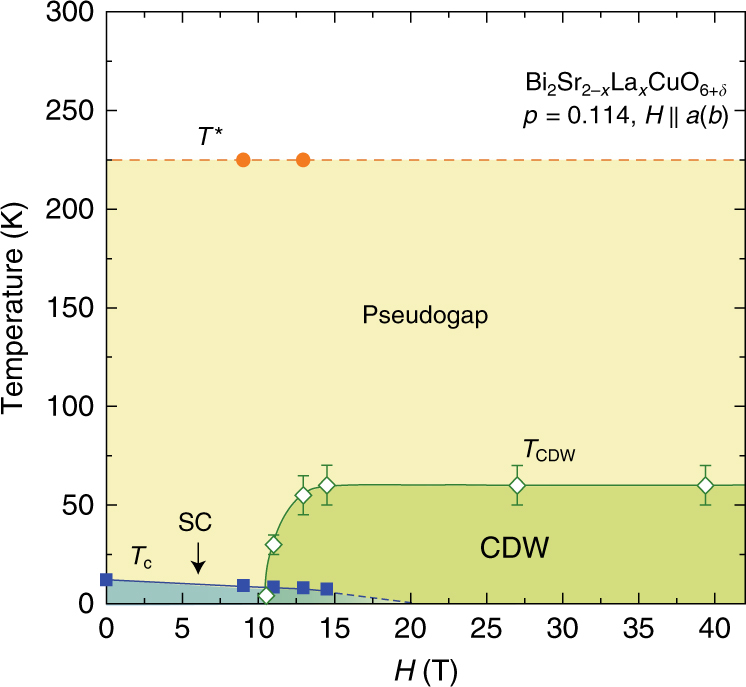
*H*-*T* phase diagram for underdoped Bi2201. The T* is the pseudogap temperature determined from the spin-lattice relaxation rate results. Error bars represent the uncertainty in defining the onset temperature *T*_CDW_

Figure 3 shows the *H*−*T* phase diagram for *p* = 0.114. Remarkably, the long-range CDW state in Bi2201 emerges at a temperature far above *T*_c_, in contrast to that in YBCO where CDW appears below *T*_c_(*H* = 0)^19,24^.

### Relationship between CDW and superconductivity

**Fig. 4:**
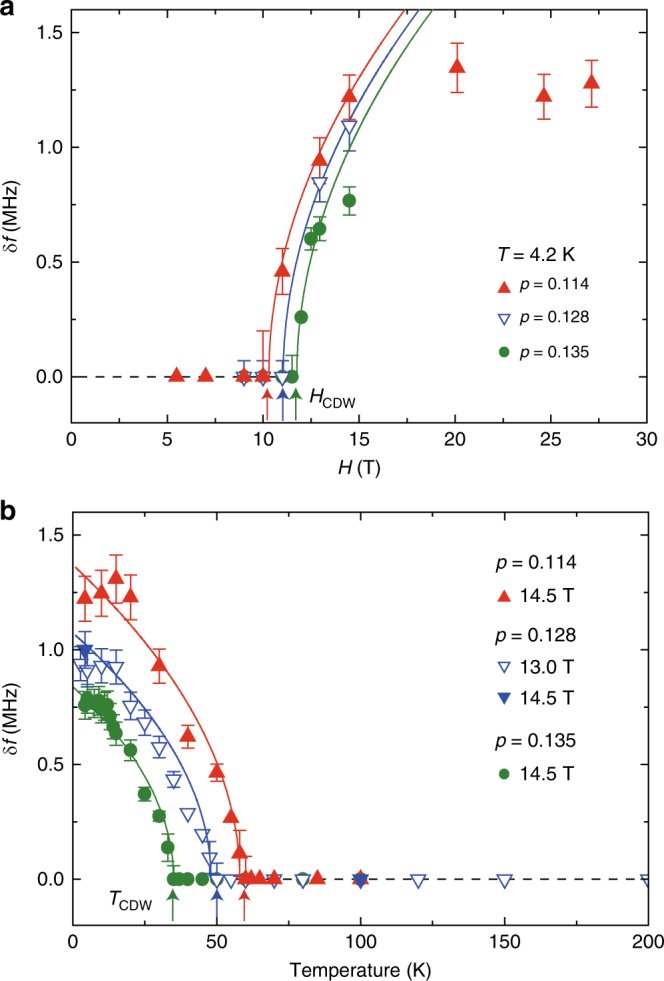
Doping dependence of *H*_CDW_ and *T*_CDW_. **a** *H* dependence of *δf* at *T* = 4.2 K. From fitting (solid curves) the data to a mean field model, *δf* ∝ (*H*−*H*_CDW_)^0.5^, the threshold field for *H*-induced CDW, *H*_CDW_ = 10.4, 11.0, and 11.8 T for *p* = 0.114, 0.128, and 0.135, respectively, was determined. **b** Temperature dependence of *δf* for the three samples, from which the CDW onset temperature *T*_CDW_ = 60, 50, and 35 K for *p* = 0.114, 0.128, and 0.135, respectively, was determined. The curves are fits to a mean field model, *δf* ∝ (*T*_CDW_−*T*)^0.5^. The dotted horizontal lines are guides to the eyes. Error bars represent the uncertainty in estimating *δf*

**Fig. 5:**
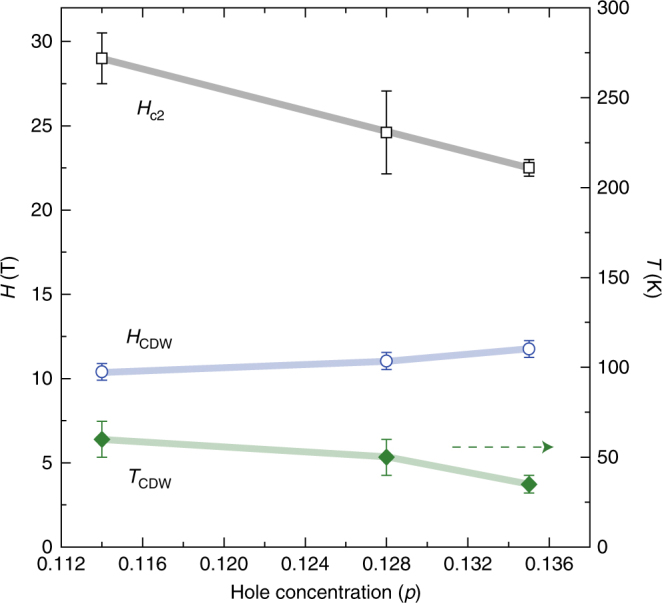
The relationship between superconductivity and the CDW in underdoped Bi2201. Doped hole concentration dependence of the upper critical field *H*_c2_, *H*_CDW_ (left axis) and *T*_CDW_ (right axis). Error bars represent the uncertainty in the fit using WHH formula to obtain *H*_c2_ (Supplementary Fig. 5) and in defining the onset field *H*_CDW_ and temperature *T*_CDW_

Figure 4a, b shows the *H*-dependence and *T*-dependence of the satellite splitting *δf* which allow us to obtain *H*_CDW_ and *T*_CDW_ for various doping levels. Figure 5 shows the hole concentration dependence of *H*_c2_, *H*_CDW_, and *T*_CDW_. The *H*_c2_ ~ 60 T for *p* = 0.162 (Supplementary Fig. 5) decreases with decreasing doping level but increasing again at *p* = 0.114. Although the previous Nernst effect study on three Bi2201 samples (*p* = 0.12, 0.16, and 0.19) did not take a closer look into the doping range as we did here^29^, our result is consistent with the results of YBCO^24^ and La_1.8−*x*_Eu_0.2_Sr_*x*_CuO_4_^29^.

The *H*_CDW_ is slightly lower than that in YBCO, suggesting that CDW has a similar energy scale across different class of cuprates. However, the relationship between *H*_CDW_ and *H*_c2_ is completely different from that seen in YBCO where *H*_CDW_ scaled with *H*_c2_. Namely, *H*_CDW_ was the lowest at the doping concentration where *H*_c2_ was the smallest there^24^, which led to the suggestion that CDW can only be seen when the superconducting state is suppressed as the vortex cores become overlapped. In the present case, however, no vortex cores are created in the CuO_2_ plane. In fact, *H*_CDW_ and *T*_CDW_ are more related with doping concentration itself as can be seen in Fig. 5, rather than with *H*_c2_. Namely, the long-range CDW order is induced more easily closer to the AF phase boundary.

## Discussion

In this section, we discuss possible CDW form and the implication of the phase diagram we found. First, the result can be understood by an incommensurate 1-dimensional (1D) long-range CDW as follows, as the situation is similar to that observed at the in-plane Cu^2F^-site located below the oxygen-filled CuO chain in YBCO^19^. For an unidirectional CDW state, the wave modulation causes a spatial distribution in the electric field gradient (EFG) and thus the NQR frequency so that *ν* = *ν*_Q_ + *δν*cos(*ϕ*(*X*))^28,30^, where *X* ( = *a* or *b* axis) is the modulation direction. The NMR spectral intensity (*I*(*ν*)) depends on the spatial variation of *ϕ*(*X*) as *I*(*ν*) = 1/(2*π*d*ν*/*dϕ*). For an incommensurate order, *ϕ*(*X*) is proportional to *X*, so that the NMR spectrum shows an edge singularity at *ν* = *ν*_Q_ ± *δν*, as $I(\nu ) = 1/\left( {2\pi \delta \nu \sqrt {1 - \left( {\left( {\nu - \nu _Q} \right)/\delta \nu } \right)^2} } \right)$ ^28,30^. By convoluting a broadening function, a two-peak structure can be reproduced. In such case, the quantity 2*δν* corresponds to the CDW order parameter^28^. We emphasize that the value of *δp* is twice larger than that observed in YBCO^19^, indicating that a larger CDW amplitude is realized in Bi2201. This difference may arise from the different crystal structure between the two systems. YBCO is a bi-layer system while Bi2201 is single layered. When CDW has a different phase between two CuO_2_ planes, the ordering effect would be weakened or even canceled out.

It has been known that magnetic field works to induce a correlation along the CuO-chain direction and modifies the coupling between CuO_2_ bilayers in YBCO^20,21^. In the present case, the short-range CDW at zero magnetic field^12,16^ could also be modified by *H*_||_ > 10 T to be a long ranged 1D CDW along the Cu−O direction.

Second, how about a 2D-CDW case? Recent resonant X-ray scattering measurement on Bi2201 *p *~ 0.11 found a perfect 2D but local (*ξ* ~ 20 Å) CDW formation along the Cu−O bond direction with the wave vector (Q*, 0) and (0, Q*) (Q* ~ 0.26)^16,31^. On the other hand, STM measurement suggested a commensurate density-wave with the ordering vectors **q**_D*W*_ = (0.25, 0) and (0, 0.25)^32^. In either case, if such a local CDW becomes long ranged with the same ordering vectors, a spatial distribution of the NQR frequency can be written as *ν* = *ν*_Q_ + *δν*_*X*_cos(*ϕ*(*X*)) + *δν*_*Y*_cos(*ϕ*(*Y*)), where *X* = *a*-axis and *Y* = *b*-axis are the modulation directions^30^. As suggested by RXS and STM measurements, when the CDW amplitudes are equivalent *δν*_*X*_ = *δν*_*Y*_, such CDWs yield $I(\nu )\sim - {\mathrm{ln}}[(\nu - \nu _{\mathrm{Q}})/\delta \nu ]/\delta \nu$ ^30^. In this case, the logarithmic singularity appears at *δν* = 0^30^, which is different from the 1D case. It is obvious that we can not explain our experimental results with such 2D CDW. However, if the amplitude for each directions is different, $\delta \nu _{X(Y)} \gg \delta \nu _{Y(X)}$, two edge-singularities will appear^30^. It is also interesting to note that if there are CDW domains with modulations *ν*_*X*_ = *ν*_Q_ + *δν*_*X*_cos(*ϕ*(*X*)) and *ν*_*Y*_ = *ν*_Q_ + *δν*_*Y*_cos(*ϕ*(*Y*)) in each domain, the NMR lineshape will be the same as in the 1D case.

Third, a 3D CDW will not show two edge singularities in *ν*_Q_ distribution in any case^30^. Therefore, we may exclude the possibility of a 3D CDW because, being different from the bilayer YBCO, the long distance between CuO_2_ planes produces no CDW correlation along the *c*-axis in Bi2201^16^ and in our case the magnetic field is applied parallel to the CuO_2_ plane.

**Fig. 6:**
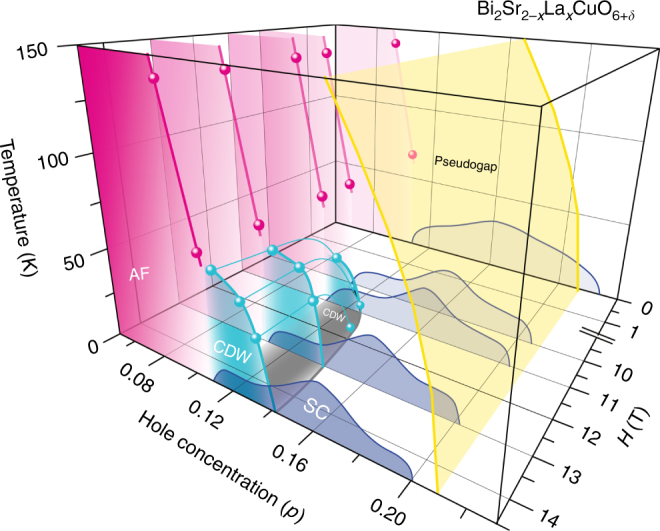
Magnetic-field evolution of the phase diagram. Hole concentration (*p*) dependence of the pseudogap temperature T*, *T*_N_, and *T*_c_, and the *p*- and *H*-dependence of *T*_CDW_ for Bi_2_Sr_2−*x*_La_*x*_CuO_6+*δ*_. Magnetic field is applied along the Cu-O bond direction ($H\parallel a(b)$). *T*_CDW_(*p*,*H*) (green and gray curves) is obtained from the results on Fig. 1f and Fig. 4. T* (yellow curve) and *T*_N_ (red curve) are from the previous works^5,18^. *T*_c_(*p*,*H*) (blue curve) is determined by the ac-susceptibility measurements using the NMR coil

**Fig. 7:**
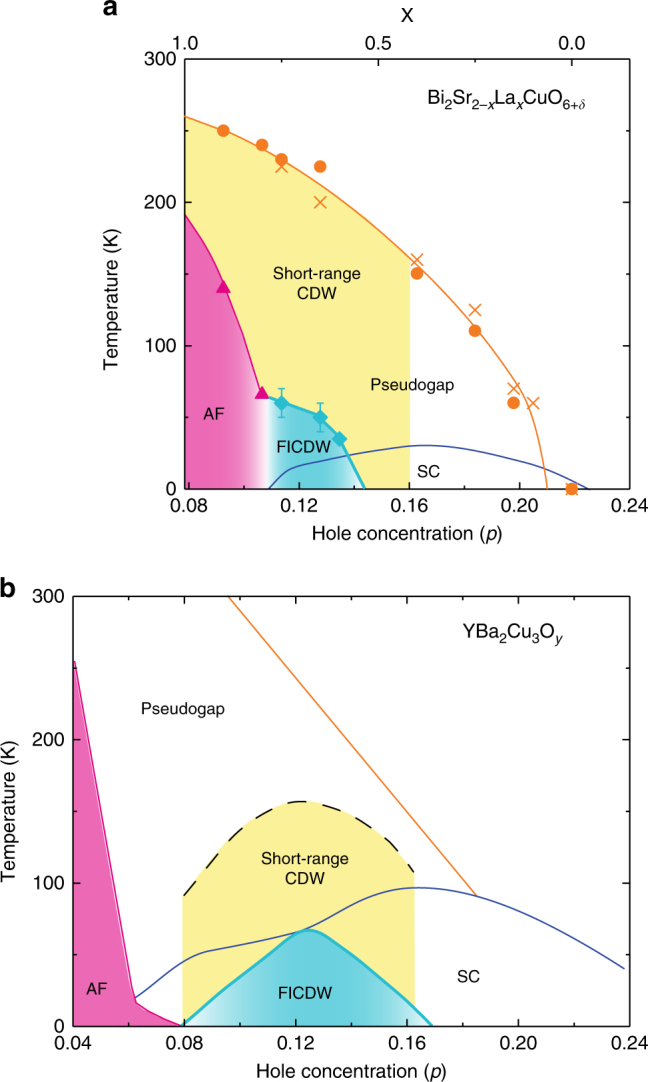
Phase diagram of Bi2201 compared with YBCO. Doping dependence of the short-range CDW reported by the X-ray measurements^2,12,16^ and the field-induced CDW (FICDW) for Bi2201 **a** and YBCO **b**. For both cases, *T*_c_ is the zero-field value. Error bars represent the uncertainty in defining *T*_CDW_. The schematic phase diagram for YBCO is from the literatures^2,19^

We now show in Fig. 6 how the long-range CDW emerges as the magnetic field is increased. As seen in the *H*-*p* plane at *T* = 4.2 K, the field-induced CDW emerges for *H*_||_ > 10 T in the underdoped regime. At such high fields, upon increasing doping, the AF state with *T*_N_ = 66 K at *p* = 0.107 changes to a CDW ordered state with *T*_CDW_ ~ 60 K at *p* = 0.114. Upon further doping to *p* = 0.162 where the pseudogap persists; however, the CDW order disappears. Although a detailed analysis is difficult, a similar field-induced CDW is also found when the magnetic field is applied perpendicular to the CuO_2_ plane (Supplementary Fig. 6). Figure 7 compares the phase diagram with that for YBCO. In YBCO, the short-range CDW sets in far below T* and the field-induced CDW (FICDW) occurs inside the superconducting dome forming a dome-like shape^19^, while in Bi2201 the short-range CDW sets in right at T* and the FICDW emerges far above the superconducting dome and coexists with superconductivity.

**Fig. 8:**
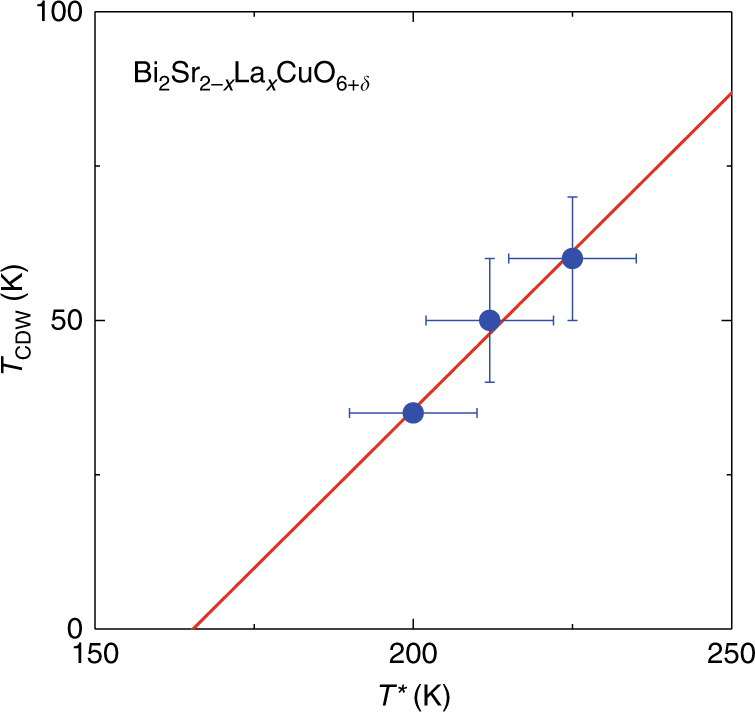
Relationship between *T*_CDW_ and T*. A plot of *T*_CDW_ vs. T* at *H* = 14.5 T. The straight line is a fitting to the data which yields a slope of 1.0 ± 0.7. Error bars represent the uncertainty in defining the two characteristic temperatures

Figure 6 reveals several important things. First and most remarkably, down-shifting the T* curve in temperature coincides with the *T*_CDW_ curve. As can be seen more directly in Fig. 8, *T*_CDW_ scales with T*. This may suggest that the pseudogap is a fluctuating form of the long-range order found in this work, but more work is needed. Very recently, a polar Kerr effect^33^ and an optical rotational anisotropy measurements^34^ suggested that a possible phase transition takes place at T*. However, we note that the probes used are ultrafast in time scale. In NMR measurements, the time scale is in the 10^−8^ s range which is much slower than the optical measurements^33,34^, and it is reasonable that T* is seen as a fluctuating crossover temperature. Second, *T*_N_ is succeeded by *T*_CDW_ beyond a critical doping level at which superconductivity emerges, pointing to the important role of charge degree of freedom in high-temperature superconductivity. However, the detailed evolution from AF to CDW order under high magnetic fields is unclear at the moment. It is a future task to clarify whether the evolution is a first-order phase transition or not. The result also calls for further scrutinies of the AF insulating phase. In fact, the entanglement of the spin and charge freedoms^3,35^ was recently found to occur already in the insulating phase itself^36^. In any case, our results show that CDW order is another outstanding quantum phenomenon that needs to be addressed on the same footing as the AF spin order. Finally, the first demonstration of the ability of using an in-plane field to tune the electronic state should stimulate more works that will eventually help to solve the problem of high-*T*_c_ superconductivity.

## Methods

### Samples

The single crystals of Bi_2_Sr_2−*x*_La_*x*_CuO_6+*δ*_ (Bi2201, *p* = 0.114 (*x* = 0.75), 0.128 (0.65), 0.135 (0.60), and 0.162 (0.40)) were grown by the traveling solvent floating zone method^37,38^. The hole concentration (*p*) were estimated previously^39^. Small and thin single-crystal platelets, typically sized up to 2 mm−2 mm−0.1 mm, cleaved from an as-grown ingot, were used. The in-plane Cu−O bond direction (*a* or *b* axis) was determined by Laue reflection. *T*_c_(*H*) is defined at the onset temperature of diamagnetism observed by ac-susceptibility measurement using NMR coil. *H*_c2_ is determined by fitting *T*_c_(*H*) to the WHH formula^40^.

### NMR

The ^63^Cu-NMR spectra were taken by sweeping the rf frequency at a fixed field below *H* = 15 T but they were taken by sweeping the field at a fixed frequency above *H* = 15 T. Measurements at *H* = 14.5 T were conducted at Institute of Physics, CAS, Beijing, and those below *H* = 14.5 T were conducted at Okayama University. High magnetic fields above *H* = 15 T are generated by the Hybrid magnet in the National High Magnetic Field Laboratory, Tallahassee, Florida. For ^63,65^Cu, the nuclear spin Hamiltonian is expressed as the sum of the Zeeman and nuclear quadrupole interaction terms, ${\cal H} = {\cal H}_{\mathrm{z}} + {\cal H}_{\mathrm{Q}} = - ^{63,65}\gamma \hbar {\mathbf{I}} \cdot {\mathbf{H}}_0(1 + K) + (h\nu _{\mathrm{Q}}/6)[3I_z^2 - I(I + 1) + \eta (I_x^2 - I_y^2)]$, where ^63^ *γ* = 11.285 MHz/T and ^65^ *γ* = 12.089 MHz/T, *K* is the Knight shift, and *I* = 3/2 is the ^63,65^Cu nuclear spin. The NQR frequency *ν*_Q_ and the asymmetry parameter *η* are defined as $v_{\mathrm{Q}} = \frac{{3eQV_{{\mathrm{zz}}}}}{{2I(2I - 1)h}}$, $\eta = \frac{{V_{{\mathrm{xx}}} - V_{{\mathrm{yy}}}}}{{V_{{\mathrm{zz}}}}}$, with *Q* and *V*_*αβ*_ being the nuclear quadrupole moment and the electric field gradient (EFG) tensor^41^. The principal axis *z* of the EFG is along the *c* axis and *η* = 0^42^. Due to ${\cal H}_{\mathrm{Q}}$, one obtains the NMR center line and the two satellite transition lines between $\left| m \right\rangle$ and $\left| {m - 1} \right\rangle$, (*m* = 3/2, 1/2, −1/2), at $\nu _{m \leftrightarrow m - 1} = ^{63,65}\gamma H_0(1 + K) + (\nu _{\mathrm{Q}}/2)(3\mathop {{cos}}\nolimits^2 \theta - 1)(m - 1/2)$ + second-order correction. The second term of the right side is the first order term in the presence of quadrupole interaction. Here, *θ* is the angle between **H** and EFG. The *T*_1_ and *T*_2_ were measured at the frequencies in the center peak (*m* = 1/2 ↔ −1/2 transition). The *T*_1_ values were measured by using a single saturating pulse and were determined by standard fits to the recovery curve of the nuclear magnetization to the theoretical function for the nuclear spin *I* = 3/2^18^. The *T*_2_ values were obtained by fits to the spin-echo decay curve of the nuclear magnetization *I*(*t*) to *I*(*t*) = *I*(0)exp(−2*t*/*T*_2_)^5^.

### Data availability

The data that support the findings of this study are available on reasonable request.

## Electronic supplementary material

Supplementary Information
