# Supplementary material for: Charge-density-wave order takes over antiferromagnetism in Bi2Sr2−xLaxCuO6 superconductors
Source: Nat Commun. 2017 Nov 2;8:1267. doi: 10.1038/s41467-017-01465-9 (PMC5668353; doi:10.1038/s41467-017-01465-9)
Supplement: Supplementary file 1 — Supplementary Information [file 41467_2017_1465_MOESM1_ESM.pdf]

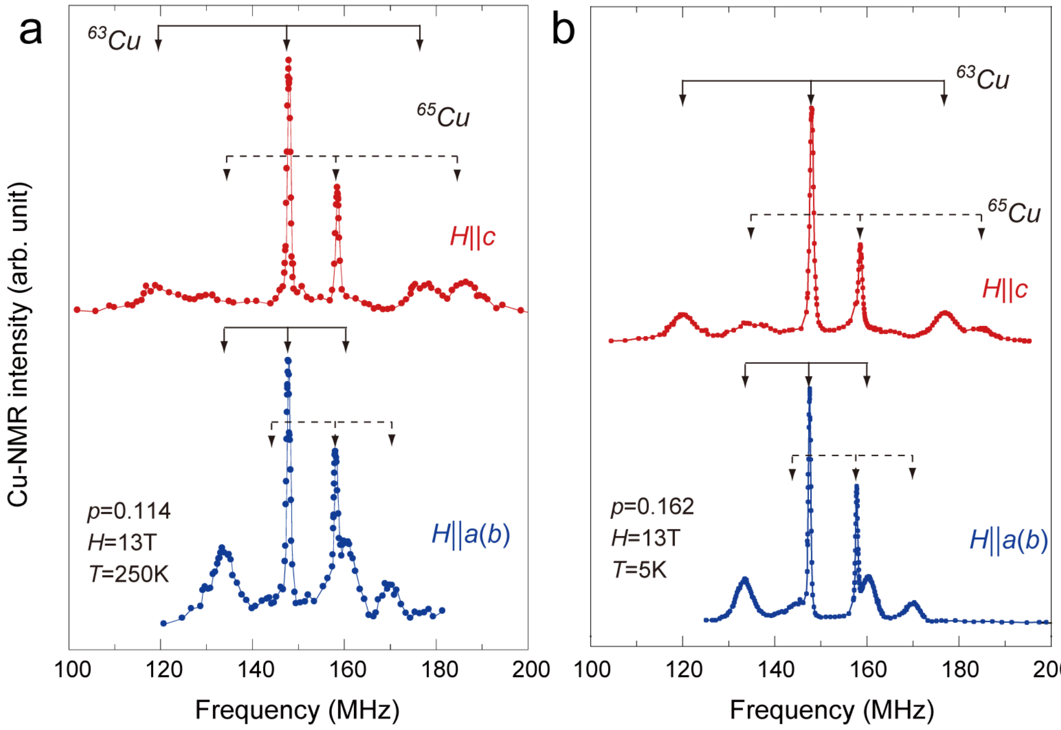

Supplementary Figure 1 | Cu-NMR spectrum. Typical ^{63,65}Cu-NMR spectrum of p = 0.114 (**a**) and 0.162 (**b**) obtained for $H \parallel c(\theta = 0^\circ)$ and $H \parallel a(b)(\theta = 90^\circ)$, respectively. The lower ^{63}Cu- NMR satellite line (3/2 ↔ 1/2 transition) peaked around 135 MHz for $H \parallel a(b)$ was used for the detection of CDW. The NQR frequency $^{63}\nu_Q$ = 26.5 MHz and 28.4 MHz for p = 0.114 and 0.162 were estimated from the angle dependence of the spectrum (see Method). From these, an empirical relation between ν_Q and hole content p, $\nu_Q = 22.0 + 39.6p$, is obtained.

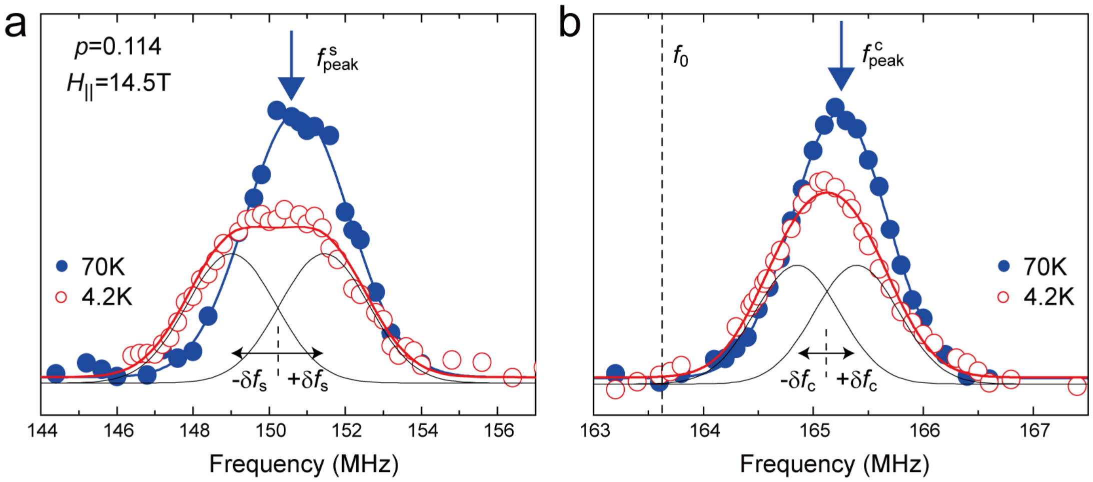

Supplementary Figure 2 | Cu-NMR spectrum analysis. Temperature dependence of the ^{63}Cu-NMR satellite (**a**) and the center (**b**) lines for p = 0.114 obtained at in-plane field $H^{||a(b)} = H_{||}$ = 14.5 T. Solid curves are Gaussian fittings to obtain the peak positions. Dotted vertical line indicates $f_0 = {}^{63}\gamma H_0 = {}^{63}\gamma H_{||}$.

Supplementary Note 1

The center and the satellite lines shift to lower frequency and become broad at T = 4.2K. The peak positions for the satellite line ($f^s_{\rm peak}$) and for the center line ($f^c_{\rm peak}$) at T = 70 K are written as follows (see Method);

$$f^c_{\rm peak}(T) = f_0 + K_{||}(T)\,{}^{63}\gamma H_0 + \frac{3{\nu_{\rm Q}}^2}{16[1+K_{||}(T)]({}^{63}\gamma H_0)} \cdots (1)\ ,$$

$$f^s_{\rm peak}(T) = f_0 + K_{||}(T)\,{}^{63}\gamma H_0 - \frac{\nu_{\rm Q}}{2} \cdots (2)$$

Here, H_0=14.5T, $f_0 = {}^{63}\gamma H_0$ = const., and $\nu_{\rm Q}$ is T-independent. The 3rd term on the right side of Supplementary Eq. (1) is the second-order correction of the quadrupole interaction. The origin of the peak shift at T = 4.2 K to a lower frequency is the reduction of $K_{||}$ due to the opening of the pseudogap and due to the superconducting transition at T_c(14.5 T) ~ 7.6 K.

Assuming that the $K_{||}$ and $\nu_{\rm Q}$ at T = 4.2 K are divided into two components as $K^{\pm}_{||}$ and $\nu^{\pm}_{\rm Q}$, respectively, we obtain the following simultaneous equations to reproduce the center and the satellite spectrum at T = 4.2 K;

$$f^c_{\rm peak}(4.2{\rm K}) + \delta f_{\rm c} = f_0 + K^{+}_{||}\,{}^{63}\gamma H_0 + \frac{3(\nu^{+}_{\rm Q})^2}{16(1+K^{+}_{||})\,{}^{63}\gamma H_0} = 165.39{\rm MHz} \cdots (3)$$

$$f^c_{\rm peak}(4.2{\rm K}) - \delta f_{\rm c} = f_0 + K^{-}_{||}\,{}^{63}\gamma H_0 + \frac{3(\nu^{-}_{\rm Q})^2}{16(1+K^{-}_{||})\,{}^{63}\gamma H_0} = 164.85{\rm MHz} \cdots (4)$$

$$+\delta f_{\rm s} = (K^{+}_{||} - K^{-}_{||})\,{}^{63}\gamma H_0 / 2 + (\nu_{\rm Q} - \nu^{-}_{\rm Q})/2 = 1.22{\rm MHz} \cdots (5)$$

$-\delta f_{\mathrm{s}} = (K_{\parallel}^{-} - K_{\parallel}^{+})^{63}\gamma H_0 / 2 + (\nu_{\mathrm{Q}} - \nu_{\mathrm{Q}}^{+}) / 2 = -1.22\mathrm{MHz} \cdots (6)$ with ν_{Q}= 26.5 MHz (see Supplementary Fig. 1).

Finally, we obtain $K_{\parallel}^{+}$(4.2K) = 0.483 %, $K_{\parallel}^{-}$(4.2K) = 0.333 %, ν_{Q}^{+} = 29.1 MHz, and ν_{Q}^{-} = 24.3 MHz. From Supplementary Figs. 1 and 2, we find that these values are produced by the carrier distribution δp = 0.06 $\pm$0.01 at the Cu-site due to the CDW. Error comes from the uncertainty in estimating $\pm\delta f_{s(c)}$.

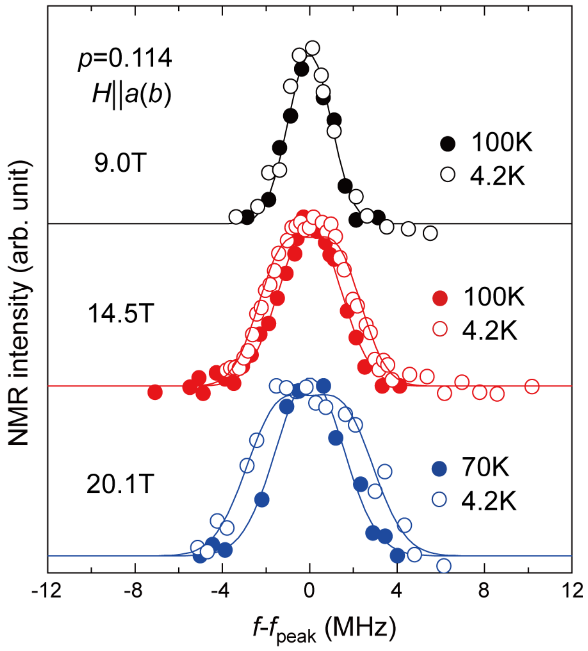

Supplementary Figure 3 | *T* and *H* dependence of the lower satellite peak. In-plane *H* dependence of the lower satellite peak for $p = 0.114$ obtained above (closed circle) and below (open circle) $T = T_{CDW}(H)$, respectively. The high-T data are fitted by a single Gaussian function, while the low-T data are fitted by a sum of two Gaussian functions. The f_{peak} is the peak frequency of the spectrum for respective magnetic field. The satellite line at $T = 4.2$ K for $p = 0.114$ is broadened at $H = 14.5$ T as for other doping concentration, which becomes pronounced above 20.1 T.

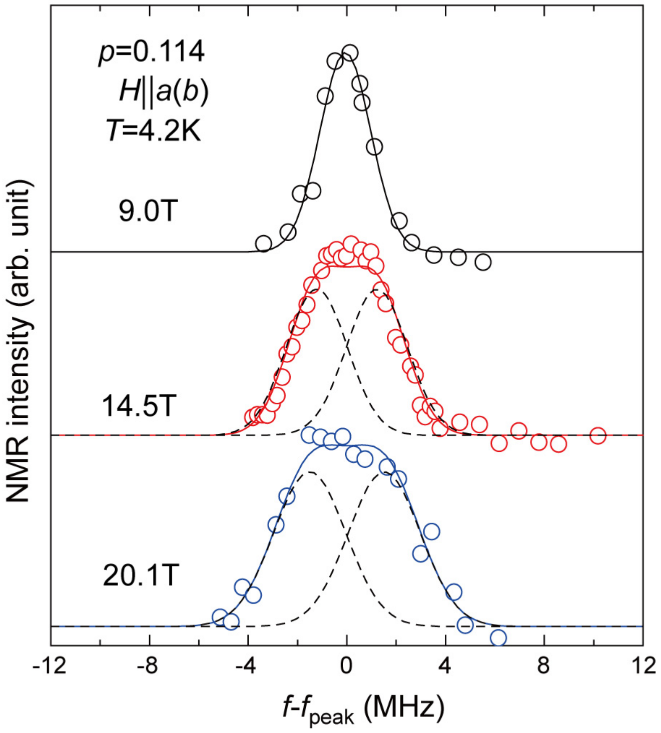

Supplementary Figure 4 | Two Gaussians fitting to obtain the *T* and *H* dependence of *δf*. In-plane *H* dependence of the lower satellite peak for p = 0.114. The f_{peak} is the peak frequency for each magnetic field. Assuming that the single Gaussian peak at 9 T splits into two peaks at $\pm\delta f(H)$ due to the CDW order above 14.5 T, a sum (solid curves) of two Gaussians centered at $\pm\delta f(H)$ (dotted curves) can fit the spectrum for H = 14.5 and 20.1 T reasonably well.

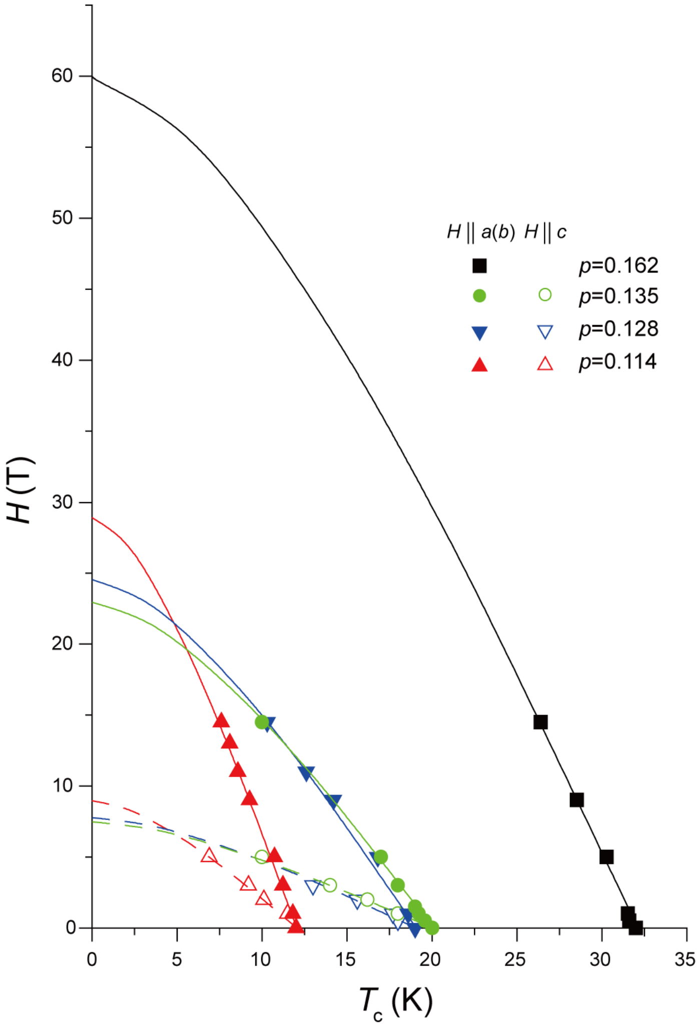

Supplementary Figure 5 | Doping dependence of H_{c2}^{χ}. *H* dependence of T_c. The upper critical field is estimated from the ac susceptibility measured by using the NMR coil. From Werthamer–Helfand–Hohenberg (WHH) formula (42), we obtain $H_{c2}^{\|a(b)}$ = 60.0, 22.5, 24.6, and 29.0 T for p = 0.162, 0.135, 0.128, and 0.114, and $H_{c2}^{\|c}$ = 7, 8, and 9 K for p = 0.135, 0.128, and 0.114 respectively. Solid and dashed curves are the fittings to WHH theory (42).

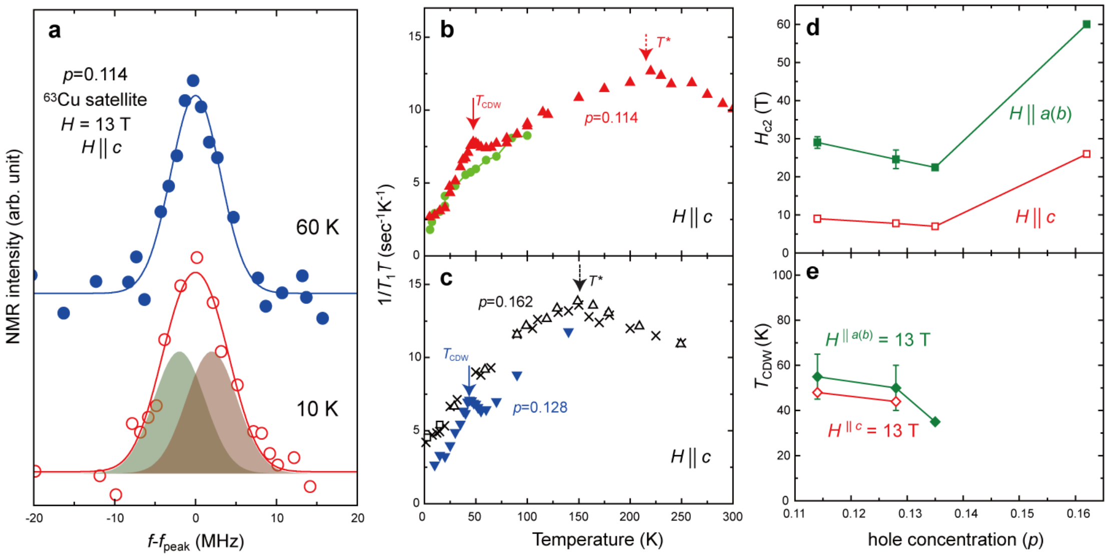

Supplementary Figure 6 |Field-induced CDW in underdoped Bi2201 for *H*||*c*. (**a**) The lower satellite line (-1/2 ↔ -3/2 transition) for $H||c$ at $T = 60$ and 10 K. The shaded two Gaussian peaks are centered at $\pm 2\delta f$ (H=13T). (**b**) Temperature dependence of $1/T_1T$ for $p = 0.114$ obtained at $H^{||c} = 9$ T (solid circles) and 13 T (solid triangle). (**c**) Temperature dependence of $1/T_1T$ for $p = 0.128$ obtained at $H = 13$ T and for $p = 0.162$ obtained at $H = 28.5$ T (crosses), 43 T (open squares), and 45 T (open triangles). The data at $H = 28.5$ T are referred from the literature (18) (**d**) Hole concentration dependence of H_{c2} obtained from the results of Supplementary Fig. 5. $H_{c2}{}^{||c} = 26$ T for $p = 0.162$ are determined by previous Knight shift measurement (5,27). (**e**) Hole concentration dependence of T_{CDW} for $H^{||a(b)} = 13$ T and $H^{||c} = 13$ T, respectively.

Supplementary Note 2

Supplementary Fig. 6a shows the temperature dependence of the satellite line for $H = 13$ T applied parallel to the c-direction for $p = 0.114$. Since the line width is much broader than the cases of in-plane fields (see Supplementary Fig. 1), the splitting due to CDW is unclear although the splitting of the satellite line for $H||c$ direction is twice larger than that for $H||a(b)$ direction (see Supplementary Fig. 1). Thus, we determine T_{CDW} by Cu-nuclear spin lattice relaxation rate ($1/T_1$). As seen in Supplementary Fig. 6b and 6c, $1/T_1T$ shows a peak at T_{CDW} under $H = 13$ T, which is absent at $H = 9$ T. Furthermore, we also find a peak in the temperature dependence of $1/T_1T$ for $p = 0.128$, although the peak is absent for $p = 0.162$ even at $H = 45$ T. This situation is the same as observed in $H||a(b)$ direction (Fig. 2d). It is quite common that $1/T_1T$ shows a peak at T_{CDW} (30). The obtained p-dependence of H_{c2} and T_{CDW} is plotted in Supplementary Fig. 6d and 6e with the result for the in-plane field $H = 13$ T. Our results indicate that the field-induced CDW in Bi2201 show anisotropy with respect to the field direction, although not as strong as superconductivity.

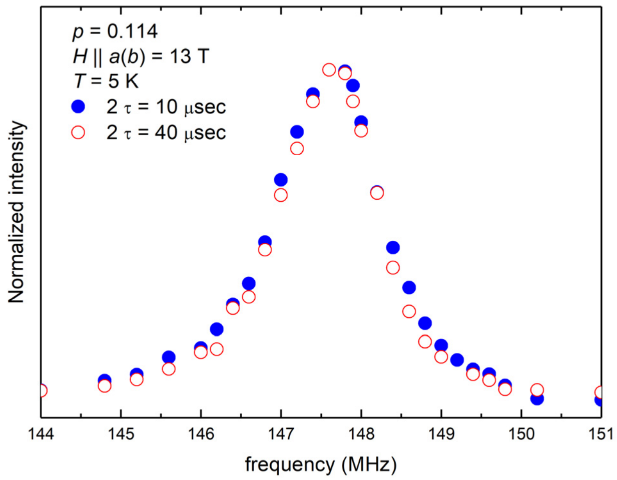

Supplementary Figure 7 | 2τ dependence of the center line spectrum. The center line (-1/2 ↔ -1/2 transition) for $H \| a(b)$ at H = 13 T and T = 5 K. The normalized spectrum is exactly the same for 2τ = 10 and 40 μsec. Here, τ is a separation of the $\pi/2$ to π pulses to obtain ^{63}Cu spin echo. The 2τ dependence of the satellite line indicates the T_2 in whole spectrum is exactly homogeneous in Bi2201.This is because the Cu-NMR site in Bi2201 is the only one in the CuO_2 plane. This situation is completely different from the inhomogeneity in YBCO, whose center line consists of the multiple spectra from the different Cu-sites (19).

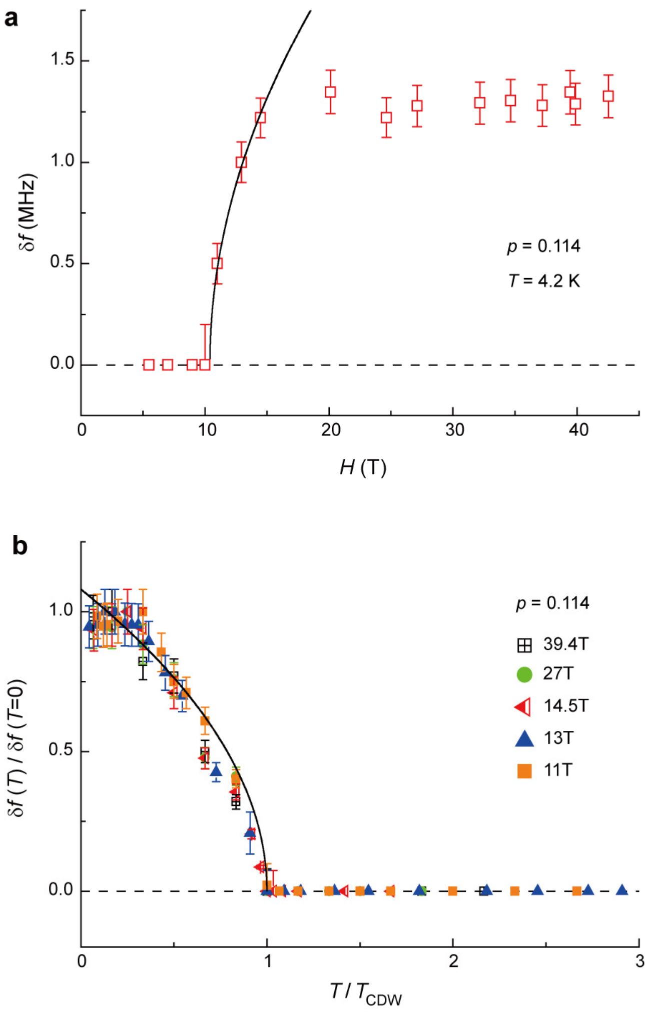

Supplementary Figure 8 |Field and temperature dependence of *δf*. (**a**) Field dependence of δf obtained at $T = 4.2$ K (same as Fig. 1e). (**b**) Temperature dependence of δf obtained under various magnetic fields. The $\delta f\,(T)$ and temperature are normalized by $\delta f\,(T)$ and T_{CDW}, respectively. The $\delta f\,(T)$ under various fields fall into a single curve, namely, scaling due to the CDW phase transition holds in $\delta f\,(T)$. Error bars represent the uncertainty in estimating δf. Solid curves are the mean field fittings, $\delta f(H) \propto (H - H_{\mathrm{CDW}})^{0.5}$ in (**a**) and $\delta f(T) \propto (1 - T / T_{\mathrm{CDW}})^{0.5}$ in (**b**), respectively.
